# Determination of Polyphenols Using Liquid Chromatography–Tandem Mass Spectrometry Technique (LC–MS/MS): A Review

**DOI:** 10.3390/antiox9060479

**Published:** 2020-06-02

**Authors:** Olalla López-Fernández, Rubén Domínguez, Mirian Pateiro, Paulo E.S. Munekata, Gabriele Rocchetti, José M. Lorenzo

**Affiliations:** 1 Centro Tecnológico de la Carne de Galicia, Rúa Galicia No 4, Parque Tecnológico de Galicia, San Cibrao das Viñas, 32900 Ourense, Spain; olallalopez@ceteca.net (O.L.-F.); rubendominguez@ceteca.net (R.D.); mirianpateiro@ceteca.net (M.P.); paulosichetti@ceteca.net (P.E.S.M.); 2 Department for sustainable food process, Università Cattolica del Sacro Cuore, Via Emilia Parmense 84, 29122 Piacenza, Italy; gabriele.rocchetti@unicatt.it; 3 Área de Tecnología de los Alimentos, Facultad de Ciencias de Ourense, Universidad de Vigo, 32004 Ourense, Spain

**Keywords:** LC–MS/MS, electrospray ionization, analytical methods, anthocyanins, flavonols, phenolic compounds

## Abstract

In recent years, the consumption of polyphenols has been increasing, largely due to its beneficial effects on health. They are present in a wide variety of foods, but their extraction and characterization are complicated since they are mostly in complex matrices. For this reason, the use of selective, sensitive, and versatile analytical techniques such as liquid chromatography coupled to tandem mass spectrometry (LC–MS/MS) is necessary. In this review, the most relevant studies of the last years regarding the analysis of polyphenols in different matrices by comprehensive LC–MS/MS are discussed. Relevant steps such as extraction, sample purification, and chromatographic analysis methods are emphasized. In particular, the following methodological aspects are discussed: (a) the proper selection of the extraction technique, (b) the extraction and elution solvents, (c) the purification step, (d) the selection of both stationary and mobile phases for the chromatographic separation of compounds, and (e) the different conditions for mass spectrometry. Overall, this review presents the data from the most recent studies, in a comprehensive way, thus providing and simplifying the information of the great variety of works that exist in the literature on this wide topic.

## 1. Introduction

**Figure 1:**
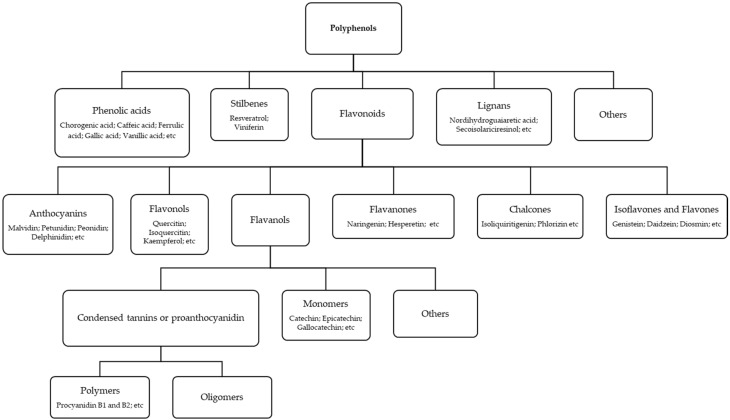
Polyphenols classification based on the number of phenol rings and their structural elements.

Polyphenols are plant secondary metabolites that are found in a wide variety of foods [1,2,3]. These natural compounds constitute a group of molecules that are divided according to their chemical structure [2,4,5], although they can also be classified by their source of origin, natural distribution or biological function. In particular, according to their chemical structure, they can be classified into different groups, as function of the number of phenol rings contained and the structural elements that bind these rings [2], as can be seen in the Figure 1.

The most common classification of polyphenols include five main classes, namely phenolic acids, stilbenes, flavonoids, lignans, and others [5,6,7]. In nature, the most abundant group of phenolic compounds are flavonoids; this is because the phenolic compounds in plants are mainly synthesized through the phenylpropanoid pathway [5,8]. Flavonoids are characterized by a phenyl benzo(c) pyrone-derived structure consisting of two benzene rings linked to a heterocyclic pyran or pyrone [9,10]. In general, they are found in a glycosylated form although they may also occur in their free form (aglycones) or polymerized [10,11]. The flavonoids are divided into anthocyanins, flavonols, flavanones, chalcones, isoflavones, flavones, and flavan-3-ols according to the degree of hydroxylation and the degree of polymerization [12]. Flavonoids can be found in vegetables (red onions, celery), cereal (buckwheat, beans), fruits and fruit by-products (apples, grapes, cherries, red wine, cherry tomatoes), spices and herbs (rosemary, oregano) [10].

Phenolic acids are derivatives of benzoic acid and cinnamic acid characterized by a high antioxidant activity, and constitute about one-third of the phenolic compounds in the human diet [5,13]. They are mainly found in strawberries, grape juice, pomegranate juice, pear, apple, lemon, and peach, among others. On the other hand, a minority group of polyphenols is represented by the stilbenes. These compounds are present in low quantities in the human diet and are characterized by a 1,2-diphenylethylene backbone. They can be found in grapes, berries, peanuts, or red wine [14]. The last group of polyphenols is the lignans that are formed from two units of a phenylpropane derivative. Overall, there are two major classes of lignans, namely the dibenzylbutane lignans and the furofuran lignans. Lignans can be found in rye, wheat, onions, citrus fruits, etc.

In recent years, numerous studies have shown that the consumption of polyphenols in the diet provides numerous health benefits. This is largely due to the antioxidant properties that help to prevent various diseases associated with oxidative stress [1,15,16]. Studies like those of Scalbert et al. [17] and Seo et al. [3] demonstrated that the antioxidant activity of plant polyphenols can retard the development of diseases such as cancer and cardiovascular and neurodegenerative diseases [3,18]. 

Besides the health implications, there is a growing interest in the use of new natural additives in food industry [19,20,21]. It is well known that oxidative reactions are the main non-microbial cause of food quality deterioration [22]. However, consumers are concerned about the diet–health relationship, and demand healthy and natural foods, forcing manufacturers to limit the use of synthetic antioxidants in food formulation. Thus, the use of polyphenol-rich extracts as synthetic additives replacers was an important strategy for food manufacturers [23,24,25].

However, the extraction and characterization of phenolic compounds in plant matrices are complex, since the phenolic compounds can be found in simple or highly polymerized structures, which can also form complexes with various other plant-matrix components. In this regard, many polyphenols are often associated with sugar moieties [2]. Thus, the use of different methods of extraction combined with proper solvents characterized by different polarities are strongly required to recover them [26]. According to Naczk and Shahidi [27], the extraction of phenolic compounds in plants is influenced by several factors. For example, some phenolic compounds are very photosensitive, as a result, rapid extraction methods are necessary to avoid the degradation of them [28]. Liquid–liquid extraction (LLE) and solid–liquid extraction (SLE) followed by a stage of concentration and purification are the most widely used methods to make a selective extraction of phenolic compounds from various matrices [2,3,29,30,31,32].

On the other hand, the most used technique for the quantification of polyphenols is UV spectroscopy due to its simplicity and low cost. However, this technique only gives an estimation of the total phenolic content and it does not separate the compounds individually. Nowadays, the liquid chromatography with diode array detector (LC–DAD) is employed for the individually separation and quantification of phenolic compounds. Nevertheless, the main limitation that presents this detector is that the compound identification is only by retention time and UV spectra. Thus, standards need to be used to correctly identify the compounds. Additionally, it may present other limitations like low detection and quantification limits in complex samples. To overcome this problem, in recent years, the use of liquid chromatography coupled to tandem mass spectrometry (LC–MS/MS) has been increasing in order to characterize the polyphenol-rich extracts. In addition, LC–MS/MS was able to achieve noise reduction and sensitivity improvements by exploiting multiple reaction monitoring (MRM) scan mode [1,28,29,33,34,35,36,37,38]. Besides, in the last years, high-resolution LC–MS and LC–MS/MS approaches coupled with multivariate statistics have been widely used to realize the so-called "metabolomic profiling" of plant foods for human nutrition. These metabolomics-based techniques (both targeted and untargeted) require minimal sample preparation and can offer a better overview regarding the polyphenol composition of a matrix under investigation, thus evaluating its bioactivity and nutraceutical potential [39,40,41]. With this in mind, the main objective of the present review is to explore the different extraction techniques, purification, separation and identification, and quantification of polyphenols by liquid chromatography-tandem mass spectrometry (LC–MS/MS). Additionally, the authors present the data from the most recent studies, in a comprehensive way, providing complete information about the main analytical parameters.

## 2. Extraction and Clean-Up Procedures

For the purpose of obtaining the good recoveries and low detection and quantification limits in the analysis of polyphenols by LC–MS/MS, the extraction and clean up stages are very important. Although these compounds have been studied extensively, there is still no common technique for their isolation.

### 2.1. Extraction

Extraction is an important step in the isolation and identification of phenolic compounds. The liquid–liquid extraction (LLE) or solid–liquid extraction (SLE) are the most commonly used and simplest extraction techniques for the isolation of phenolic compounds. Several researchers in the literature focus on the extraction and analysis of polyphenols in different plant materials such as wine, tea, oil, herbs, and fruits among others (Table 1).

Solvent extractions consist of a direct extraction of polyphenolic compounds in samples (previously ground, dried, or lyophilized) by soaking the samples with the extraction solvent [42]. Polyphenol extraction in samples takes place by stirring (vortexes, orbital shaker, automatic shaker, or ultrasonic bath) during a determinate time at controlled temperature.

The efficiency of extraction process can vary in function of process conditions [27,43,44]. Phenolic compounds extraction is influenced by several factors, such as chemical nature of phenolic compound, extraction method, sample particle size, extraction solvent, pH, and temperature, among others [43,44]. Many authors have studied the influence of these factors on the efficacy of the extraction process [45].

The most important factor is the choice of the correct extraction technique. Wang et al. [29] compared the liquid–liquid extraction (LLE) and the solid-phase extraction (SPE) for the extraction of 15 polyphenols (eight phenolic acids, three flavonols, and four anthocyanins) in rice wine. In this work, the authors concluded that LLE is more effective for phenolic acids and flavonoids, whereas for anthocyanin extraction by SPE is better. Similarly, Bajckacz [31] compared solid–liquid extraction with the QuEChERS (Quick, Easy, Cheap, Effective, Rugged, and Safe) method for the extraction of flavonoids and phenolic acids in plant material. QuEChERS is a novel extraction method created to avoid the use of large solvent volumes and to reduce the purification times [46]. However, worse results were obtained with this method than with traditional SPE extraction.

In general, as can be seen in Table 1, the most common extraction solvents for polyphenol extraction are methanol, acidified methanol, or combinations of methanol–water. The choice of the solvent is also vital for an optimal extraction. In fact, in 2018 Bajkacz et al. [31] observed how the polyphenols content varied as a function of solvents and extraction times used. These authors studied the flavonoid and phenolic acids content in different plant materials (lucerne, goldenrod, phacelia, buckwheat, licorice, and lavender) by solid–liquid extraction using water, ethanol, methanol, or combination of them as extraction solvents. In this study a considerable increase of the mean content of extracted polyphenol can be observed when methanol or ethanol were used instead of water. For example, in licorice plants an increase in the mean of polyphenols from 207 to 5566 ng/g was observed when water or methanol, respectively, was used. Methanol was the best extraction solvent followed by ethanol or combinations of them. Nevertheless, in food industry ethanol is preferred due to the methanol toxicity [31,43]. The chemical nature of the matrix constituents and the polarity of the extraction solvent influence the phenolic compound solubility. In the case of highly polar phenolic compounds, the extraction with pure organic solvents is not effective. Consequently, the addition of solvents with higher polarities are necessary to increase the overall polarity of the solvent mixture [47]. Phenolic acids or highly glycosylated flavonoids require mixtures of organic solvents with water, for example, 75% aqueous acetone [48], 80% aqueous ethanol [36], or 80% aqueous methanol [49].

**Table 1:** Different conditions for the polyphenol extraction and purification.

Matrix	Analyte	Extraction (Solvent Extraction) and Purification (Cartridge)	Recovery (%)	LOD (mg/L)	LOQ (mg/L)	Ref
Mutamba (*Guazuma ulmifolia* Lam.) fruit	Phenolic acids (*n* = 10)	SLE (1 g + 15 mL methanol/acetone/water (7/7/6, *v*/*v*/*v*), 30 min US, RT) × 3 times	-	-	-	[50]
	Flavanols (*n* = 3)					
	Flavonols (*n* = 6)					
	Flavanones (*n* = 1)					
	Flavones (*n* = 1)					
	Procyanidins (*n* = 2)					
Meiguihua oral solution	Phenolic acids (*n* = 2)	LLE (dilution 1:100 in methanol)	92.68–101.45	1.09–6.54	13.14–3269	[33]
	Flavonols (*n* = 8)		92.30–102.80	0.11–0.87	0.44–5.54	
Wheat pasta chia flour	Phenolic acids (*n* = 13)	SLE (5 g + 20 mL solvent mixture of acetone/water (4:1), 1 h shaking, RT, darkness) × 2 times		0.31–0.95	0.09–0.28	[48]
Extra-virgin olive oils, olive fruits and pomaces	Phenolic acids (*n* = 3)	LLE or SLE (2.5 g + 5 mL ethanol/water, 80/20, *v*/*v*, 10 min US at 21 °C)		10.0–30.0	3.0–10–0	[36]
	Flavonols (*n* = 2)			10.0	3.0	
	Flavones (*n* = 1)			10.0	3.0	
	Flavanones (*n* = 1)			10.0	4.0	
*Artemisia campestris*	Phenolic acids (*n* = 3)	SLE (1 g + 10 mL ethanol/water 8/2 *v*/*v*, 30 min US at RT)				[51]
	Flavonols (*n* = 2)					
	Flavones (*n* = 1)					
Residual brewing yeast	Phenolic acids (*n* = 6)	MSPD (0. 10 g + 10 mg TiO_2_ nanoparticles (NPs) and 0.1g diatomaceous earth); mixed 2 min in mortar, add 2 mL ethanol/water 60/40 *v*/*v*, 1 min vortex				[1]
	Flavonols (*n* = 3)					
	Flavanones (*n* = 1)					
	Flavones (*n* = 1)					
*Achyrocline satureioides*	Phenolic acids (*n* = 1)	SLE (methanol) and SPE C-18	-	-	-	[10]
	Flavones (*n* = 1)					
	Flavonols (*n* = 3)					
*Fragaria ananassa* cv. *Camarosa* fruits	Anthocyanins (*n* = 6)	SLE (10 g + 10 mL methanol/formic acid (97/3, *v*/*v*), 30 s US, RT and 16 h, orbital shaking, RT) × 2 times and SPE (Oasis MCX cartridges eluted with 15 mL methanol)	-	0.14	0.48	[32]
	Flavonols (*n* = 1)			0.4	1.5	
Sweet lupin seed	Phenolic acids (*n* = 4)	SLE (2 g + 10 mL methanol/water 80/20 *v*/*v*, 10 s vortex, 2 h orbital shaking) × 2 times	97.61–100.76	0.001–0.035	0.004–0.119	[49]
	Isoflavones (*n* = 1)		97.89	0.004	0.013	
	Flavones (*n* = 1)		97.72	0.030	0.100	
	Flavanonol (*n* = 1)		104.38	0.019	0.065	
Lucerne, goldenrod, phacelia, buckwheat, licorice, and lavender	Phenolic acids (*n* = 13)	SLE (2.5 g + 10- or 20-mL methanol, automatic shaker 5 h at 900 rpm) and SPE (C18, 6 mL, 500 mg, eluted with 6 mL methanol)	58.9–95.5	-	0.0004–0.02	[31]
	Flavonols (*n* = 3)		72.6–79.3	-	0.0004–0.0008	
	Isoflavones (*n* = 2)		56.3–79.5	-	0.0004	
	Flavanones (*n* = 10)		49.1–95.2	-	0.0004–0.0008	
Commercial herbal dietary supplements	Flavanones (*n* = 6)	SLE (0.2–0.7 g + 5 mL methanol, 30 min US mL) × 3 times	64.6–76.8	0.00016–0.00025	0.0005–0.0008	[38]
	Isoflavones (*n* = 2)		72.4–81.9	0.00022–0.00025	0.0007–0.0008	
	Flavonols (*n* = 2)		74.6–80.3	0.00022–0.00033	0.0007–0.001	
Brown seaweed	Phenolic acids (*n* = 2)	SLE (5 g + methanol/water 60/40 v/v; under nitrogen atmosphere for 2 h; 40 °C, 100 rpm shaker incubator) and SPE (C18) eluted with 15 mL methanol with 0.1% HCl	99.3–104.2	0.26–0.73	0.77–2.50	[2]
	Flavonols (*n* = 2)		97.2–98.4	0.51–0.57	1.79–1.82	
	Anthocyanins (*n* = 1)		97.7	0.34	1.14	
Fruits from *Firmiana Simplex* (L.)	Phenolic acids (*n* = 2)	SLE (500 g + 2 L methanol at RT) × 4 times	-	-	-	[52]
	Flavanols (*n* = 2)		-	-	-	
	Flavones (*n* = 1)		-	-	-	
	Lignans (*n* = 1)		-	-	-	
Red grapes	Anthocyanins (*n* = 3)	SLE (0.8 g+ 1 mL methanol (1% formic acid)/water) 60/40, *v*/*v*; 72 °C, 100 min 500 rpm	-	0.003–0.006	0.010–0.021	[34]
	Phenolic acids (*n* = 3)		-	0.002–0.0040	0.006–0.135	
	Flavonols (*n* = 4)		-	0.003–0.342	0.010–1.140	
*Connarus perrottetti* var. *angustifolius*, *Cecropia obtusa*, *Cecropia palmata*, and *Mansoa alliacea*	Phenolic acids (*n* = 4)	SLE (0.2 g + 70% hydroethanolic, butanol or ethyl acetate, 4 h, US, RT)	97.6–104.7	0.3–0.7	0.8–1.0	[28]
	Flavonols (*n* = 2)		88.2–94.6	0.4–0.6	0.8–2.4	
	Flavanols (*n* = 1)		83.8	1.7	2.8	
*Lablab purpureus* (L.) sweet pods	Anthocyanins (*n* = 5)	SLE (0.1% HCl in methanol/water; 35/65 *v*/*v*)	-	-	-	[53]
*Syringa vulgaris* L. flowers and fruits	Oleuropein	SLE (0.020 g lyophilized sample + 5 mL methanol; stirred 4 h, 200 rpm at RT) × 3 times	101.0	0.0021	0.0068	[37]
	Acteoside		97.4	0.0008	0.0024	
	Rutin		94.9	0.0003	0.001	
Tea	Flavanols (*n* = 12)	SLE (1 g sample dried + 100 mL hot water, 3 min, mild stirring) × 5 times	65–115	0.12–223.70	0.40–745.60	[54]
				(pg/injection)	(pg/injection)	
*Euphorbia supina*	Phenolic acids (*n* = 3)	SLE (10 g sample lyophilized + 200 mL ethyl acetate, 20 h, 80 °C)	79.6–102.8	0.030–0.142	0.102–0.473	[30]
	Flavonols (*n* = 2)	SPE (silica gel (3 × 1.7 cm i.d.), eluted with 25 mL methanol/dichloromethane 1/5 v/v)	76.1–100.0	0.028–0.037	0.094.0.125	
Black rice wine	Phenolic acids (*n* = 8)	LLE (5 mL sample pH 2.0 + 5 mL ethyl acetate, 1 min, vortex) and SPE (Oasis HLB (200 mg, 6 mL), eluted with 8 mL methanol with 0.1% of HCl)	74–103.0	0.008–0.003	0.027–0.100	[29]
	Flavonols (*n* = 3)		63.0–81.0	0.008–0.024	0.027–0.080	
	Anthocyanins (*n* = 4)		62.0–70.0	0.010–0.020	0.030–0.060	
*Scutellaria baicalensis*	Flavones (*n* = 9)	SLE (10 g lyophilized sample + 200 mL methanol, 24 h, 50 °C) and SPE (silica gel, eluted with 50 mL methanol/dichloromethane, 1/5, v/v)	82.3–107.7	0.007–0.044	0.021–0.133	[3]
	Flavanones (*n* = 5)		80.1–99.0	0.11–0.76	0.025–0.145	
	Phenolic acids (*n* = 2)		104.3–101.7	82.3–101.7	0.004–0.010	
	Flavonols (*n* = 1)		87.6	0.017	0.052	

US: ultrasound extraction; RT: room temperature; SLE: solid–liquid extraction; LLE: liquid–liquid extraction; MSPD: matrix solid-phase dispersion; SPE: solid-phase extraction.

The pH is another important factor that influences the extraction of phenolic compounds. It depends on the nature of the compounds to be extracted and the sample. In general, it is necessary to use low pH in the solvent extraction in order to prevent the oxidation of phenolic compounds. Acidification of the solvent increases the ability to extract phenolic compound. This fact is due to the addition of acid control charge, which greatly influences polyphenol extraction. Table 1 shows several works using methanol acidified with formic acid or hydrochloric acid for the extraction.

Polyphenols extraction is also affected by contact time and liquid–solid or liquid–liquid ratio [43]. In the literature the extraction time of polyphenols is very variable ranging from a few minutes to several hours (Table 1). Bajkacz et al. [31] studied the influence of two extraction times (2 or 5 h) over the content of polyphenols in plant material extracts observing an increase in polyphenols content with longer extraction times. Extraction cycles are usually repeated various times and the obtained extracts are further mixed to increase the extraction efficiency. Bajkacz et al. [31] also compared the efficacy between a single extraction or various extraction cycles. They observed that various extraction cycles improve the extraction efficiency of polyphenol compounds from plant materials compared to one extraction cycle with the same solvent [31]. However, an excessive increase in the extraction time may cause degradation of polyphenols mainly due to oxidation [44,55].

Finally, another relevant parameter is the temperature. It is known that high temperatures improve extraction efficiency since heat increases the permeability of cells, the diffusion coefficients, the solubility, and mass transfer rate of the compounds studied. It also modifies the solvent properties making it less viscous, leading to an increase of polyphenol transference to the solvent [44,56]. In 2018 Carres et al. [57] selected five different temperatures between 25 and 85 °C to study the effect of temperature over the polyphenols extraction yield in red grapes. Generally, they observed how an increase in temperature meant an increase in the yield of the extraction process. However, in the case of anthocyanins, the yield was increased until 70 °C. At higher temperatures (85 °C) the performance dropped slightly in comparison with the other extraction temperatures, probably due to the thermosensitivity of anthocyanins (Table 1). In the same manner temperatures below 40 °C were not effective for polyphenol extraction [58,59,60].

All extraction methods involve a stirring stage that can be mechanical stirring, vortex, or ultrasound treatment. The latter one is considered the most effective method to isolate polyphenols [61]. This fact is due to the ability of ultrasound treatment to damage cell walls, allowing the release of intracellular compounds and increased the solute/solvent contact [62]. Moreover, nowadays, improvements in ultrasound technology grant the opportunity to extract bioactive compounds with economic advantages [62]. Adjé et al. [63] evaluated the efficiency of agitating mode (ultrasound-assisted procedure or mechanical stirring) for anthocyanin, flavonols, and phenolic acid extractions from *Delonix regia* tree flowers. The results obtained showed that total polyphenol content was similar with both stirring modes or slightly higher for mechanical stirring. However, the ultrasound procedure shortened maceration time up to three times. This is important considering that shorter extraction times may avoid compound degradation. In the same way, Altemimi et al. [64] showed this for spinach extracts, demonstrating that the content in total polyphenols was four times higher with ultrasound compared to conventional agitation. This aspect is related to the fact that ultrasound-assisted extraction involves the formation of cavitation bubbles, which assist the release of the vegetable content, thus increasing the mass transfer [65].

### 2.2. Clean-Up

Purification, fractionation, and concentration of the sample are of great importance for polyphenol analysis [44]. Generally, the solvent extraction implies the co-extraction of other non-phenolic substances, such as sugars, glycosides, organic acids, fats, alkaloids, terpenoids, waxes, and pigments [56,66,67]. Hence, one additional step of clean-up prior to liquid chromatography analysis is necessary, with the aim of removing these substances and avoiding possible interferences. 

The extraction in solid phase (SPE) and liquid–liquid extraction (LLE) are the most employed techniques in clean-up procedures [46,68]. In liquid–liquid extraction the use of non-polar solvents contributes to avoiding lipid interferences of the matrix in LC–MS/MS analysis. Most authors use solvents such as hexane, chloroform, dichloromethane, or petroleum ether for defatting the samples [46,56]. However, since the LLE technique requires large amounts of solvents in this process, nowadays, SPE is used as an alternative for the purification of polyphenols [46].

In SPE, the target compounds are retained in a sorbent and then are eluted with an adequate solvent (methanol, ethanol, ethyl acetate). The SPE process is rapid, economical, and simple [69] and allows the purification and concentration of polyphenols at once. In liquid samples SPE is used as an extraction technique more than a clean-up step. In the last few years, as shown in Table 1, different SPE cartridges were used to remove interfering compounds from several extracts, such as C18 [2,31], Oasis MCX [32], HLB [29], or silica gel [3,30] as stationary phases. Prior to the loading of the sample into the cartridges, it is necessary to precondition them. The most used conditioning solvents are water, methanol, and their combinations [2,29,31]. Bajkacz et al. [31] conditioned the C18 SPE columns (500 mg sorbent mass) with 6 mL of methanol and 6 mL of acidified water, while Rajauria [2] conditioned C18 SPE (10 g sorbent mass) with 60 mL of methanol and 60 mL of water. Similarly, Wang [29] also conditioned both, C18 (500 mg sorbent mass) and Oasis HLB (200 mg sorbent mass) with 2 mL of methanol, followed by 2 mL of water. After loading the sample into the cartridge, the co-extracted substances such as sugars, acids, and other polar compounds were eluted from the SPE columns with acidic water [2] or water [29]. Thereby, in order to remove the more hydrophilic compounds Martinechi et al. [35] used a mixed of methanol:water (20:80, *v*/*v*). Finally, to elute phenolic compounds, it is common to use organic compounds such as methanol [31,32] or acidified methanol [2,29] (Table 1). Other authors use methanol combined with water [35] or dichloromethane [3,30]. In 2014, Wang et al. [29] studied the influence of two purification sorbents (C18 silica and Oasis HLB), as well as, the influence of different elution solvents (methanol and acidified methanol) and different elution volumes (2, 4, 6, 8, and 10 mL). Regarding retention of polyphenols, Oasis HLB sorbent (from two monomers divinylbenzene and N-vinylpyrrolidone) was more effective for both polar and nonpolar compounds. While, acidified methanol turned out to be the most suitable extraction solvent since it improved the extraction, especially in the case of anthocyanins. This is because acidic environments help the dissolution of anthocyanins [29,70].

Another alternative to traditional solid-phase extraction (SPE) is the matrix solid-phase dispersion assisted extraction (MSPD). This methodology is rapid and simple, consumes less solvent, and generates few residues. Gómez-Mejía et al. [1] in 2019 used the MSPD technique to extract and purify several polyphenols from residual brewing yeast. After extraction, polyphenols were identified and quantified by liquid chromatography coupled to a triple quadrupole analyzer (LC–MS/MS). Nevertheless, to obtain good results it was convenient to optimize several parameters such as extraction solvent, amount of sample, and stirring mode. Thus, Gomez-Mejía evaluated the selectivity and efficiency of methanol, ethanol, and ethanol-water 20:80 (*v*/*v*) and 60:40 (*v*/*v*), two different amounts of samples (0.05 and 0.10 g) and stirring mode (ultrasonic bath or vortex shaking). Among the studied solvents ethanol-water mixtures and pure methanol gave better results. A reduction of sample amount showed a decrease of the major compounds (gallic acid and naringin) and the non-detection of rutin and quercetin. In general, the best results were obtained when vortex-assisted stirring mode was used. The ultrasound bath produced reductions between 55% and 85%.

Thus, the most important parameters in extraction and clean-up procedures must be carefully selected to ensure correct extraction of phenolic compounds for a reliable identification and quantification.

## 3. Chromatographic and Mass Spectrometry Conditions

The total content of polyphenols is determined by spectrophotometric techniques that are fast, easy, and cheap, however these techniques are not able to identify phenolic compounds individually. Due to the need to identify them individually, it is required to replace these traditional methods by chromatographic analysis that provide more specific and detailed information [46,71].

Liquid chromatography (LC) is the most used technique for achieving the separation, identification, and quantification of polyphenolic compounds in different matrices. However, to date there is still no single chromatographic method capable of separating the different types of phenolic compounds. Depending on each group of compounds it is necessary to optimize the stationary phase, mobile phase, gradient elution, temperature, and flow rate. In addition, other factors such as stereochemistry, molecular weight, polarity, and degree of polymerization of polyphenols have to be taken into consideration since they affect the retention of the compounds [72,73,74,75].

Usually, separation of phenolic compounds by LC is carried out in the reverse phase (RP) mode with columns, generally packed with particles of silica bonded with alkyl chains (C8 or C18) and various mobile phases as can be seen in Table 2 [46,71,75]. In the scientific literature (Table 2), the column length varied from 10 to 250 mm in length and the internal diameter varied between 2.0 and 4.6 mm. Baranowska and Bajkacz [38] evaluated the efficacy of C8 and C18 columns and different composition of mobile phase for polyphenol determination in nine commercial herbal dietary supplements. In this study, the authors concluded that C18 was found to be more suitable as it showed better separation of analytes with satisfactory peak shapes as compared to C8.

To have more reproducible elution times and greater resolution of the peaks, column temperature is generally controlled. The used temperature values normally varied between 25 and 40 °C (Table 2). Higher temperatures also contribute to reducing the pressure of the column when high flow rates are applied and decrease the analysis time [46].

**Table 2:** Chromatographic and mass spectrometer conditions used in polyphenol analysis.

Analyte	Matrix	Chromatographic Conditions	Mass Spectrometer Conditions	Ref.
Quinic acid; danshensu; caftaric acid; caffeic acid hexoside; salvianolic acid; fertaric acid; caffeic acid; ferulic acid; salviaflaside; rosmarinic acid; salvianolic acid c; methylrosmarinate; methylquercetin	Wheat pasta chia flour	Column: Luna C18 (250 × 4.6 mm, 5 μm)	Capillary voltage: 4500 V	[48]
		Column temperature: 35 °C	Nebulizer gas: 4.0 bar	
		Mobile phase: 0.5% formic acid in water and 0.5% formic acid in methanol (*v*/*v*)	Drying gas: 8.0 L/min and 180 °C	
		Flow rate: 0.4 mL/min	Nebulizer gas: nitrogen	
		Injection volume: 40 µL	Collision gas: argon	
Gallic acid juglanin; quercetin-3-O-sophoroside; ellagic acid; quercitrin; sophoraflavonoloside; hyperoside; astragalin; isoquercitrin; avicularin	Meiguihua oral solution	Column: Hypersil Gold C18 (100 × 2.1 mm, 1.9 μm)	Capillary voltage: −4500 V	[33]
		Column temperature: 30 °C	Declustering potential: −10 V	
		Mobile phase: 0.1% formic acid in water and acetonitrile	Nebulizer gas: 60 Curtain gas: 35	
		Flow rate: 0.3 mL/min	Auxiliary gas: 50	
		Injection volume: 2 µL	Turbo gas temperature: 450 °C	
P-hydroxybenzoic acid; caffeic acid; chlorogenic acid; ellagic acid; ferulic acid; gallic acid; gentisic acid; p-coumaric acid; luteolin; protocatechuic acid; catechin; epicatechin; epigallocatechin; kaempferol; astragalin; nicotiflorin; cynaroside; naringenin; procyanidin dimer b1 y b2; procyanidin trimer c1; quercetin; hyperoside; isorhamnetin; rutin; vanillin	Mutamba (*Guazuma ulmifolia* Lam.) fruit	Column: Shimpack XR-ODS III column (150 × 2 mm, 2.2 μm)	Capillary voltage: 3.5 kV	[50]
		Column temperature: 40 °C	Heat block temperature: 300 °C	
		Mobile phase: 0.1% formic acid in water and methanol	Desolvation line temperature: 250 °C	
		Flow rate: 0.4 mL/min	Nebulizer and drying gas: nitrogen	
		Injection volume: 10 µL	Drying flow: 20 L/min	
			Nebulizing flow: 3 L/min	
			Collision induced dissociation gas: argon at 224 kPa	
Protocatechuic acid; 5-O-Caffeoylquinic acid; Quinic acid methyl ester; 3-O-Caffeoylquinic acid; Caffeic acid; 4-O-Feruloylquinic acid; Quercetin-O-glucoside; Rutin; 3,4-Dicaffeoylquinic acid; 4,5-Dicaffeoylquinic acid; 4′,7′-Dimethoxy luteolin	*Artemisia campestris*	Column: Lichrocart RP-18 column (250 × 4 mm, 5 μm)	Collision gas: argon at 10^−4^ mbar	[51]
		Column temperature: 35 °C	Nebulizer and drying gas: nitrogen	
		Mobile phase: Formic acid aqueous solution (0.5% *v*/*v*) and acetonitrile		
		Flow rate: 0.3 mL/min		
		Injection volume: 10 µL		
Caffeic, chlorogenic, p-coumaric, 3,4-dihydroxibenzoic, trans-ferulic and gallic acids, kaempferol, myricetin, naringin; quercetin; rutin	Residual brewing yeast	Column: C18 Fusion-RP (150 × 3 mm, 4 µm)	Nebulizer and drying gas: nitrogen	[1]
		Column temperature: room temperature	Flow nebulizer gas: 1.5 L·min^−1^	
		Mobile phase: 0.2% formic acid aqueous solution and methanol	Flow drying gas: 15.0 L·min^−1^	
		Flow rate: 0.50 mL/min	Collision gas: argon at 230 kPa	
		Injection volume: 20 µL	Ionization voltage: −4.5 kV	
Verbascoside; Isoverbascoside; Forsythoside A; Leucosceptoside A; Plantainoside C; Purpureaside D; Martynoside	*Aloysia polystachya*	Column: Ascentis Express C18 (100 × 2.1 mm, 2.7 µm)	Capillary voltage: −4000 V	[35]
		Column temperature: 30 °C	Drying gas: nitrogen	
		Mobile phase: 0.1% formic acid in water and 0.1% formic acid in acetonitrile	Drying gas temperature: 350 °C	
		Flow rate: 0.2 mL/min	Flow drying gas: 9 L/min	
		Injection volume: 10 µL	Nebulizing gas: 25 psi	
Dicaffeoylquinic acid isomer A and B; iIsoquercitrin; quercetin; luteolin; 3-O-methylquercetin	*Achyrocline satureioides*	Column: Luna C18 (150 × 4.6 mm, 5 µm)	Capillary voltage: 4000 V	[10]
		Column temperature: 40 °C	Nebulizer: 40 psi	
		Mobile phase: 10 mM formic acid in ultra-pure water and methanol	Dry gas flow: 9.0 L/min at temperature 365 °C	
		Flow rate: 0.3 mL/min	Drying and nebulizing gas: nitrogen	
		Injection volume: 5 µL		
Cyanidin-3-glucoside; pelargonidin-3-glucoside; pelargonidin-3 rutinoside; pelargonidin-acetylglucoside; pelargonidin-succinyl-arabinoside; pelargonidin-malonylrhamnoside; quercetin-rhamnoside	*Fragaria ananassa* var. *Camarosa* fruits	Column: Kromasil C18 (250 × 4.6 mm, 5 μm)	-	[32]
		Column temperature: 40 °C		
		Mobile phase: water/acetonitrile/formic acid (87/3/10 *v*/*v*/*v*) and water/acetonitrile/formic acid (40/50/10 *v*/*v*/*v*)		
		Flow rate: 0.8 mL/min		
Caffeic acid; chlorogenic acid; p-Coumaric acid; ferulic acid; rutin; quercetin; luteolin; naringenin; genistein	*Olea europaea* L.	Column: Kinetex biphenyl (10 × 2.1 mm, 5 μm)	Nebulizer gas: nitrogen	[36]
		Column temperature: 35 °C	Capillary voltage: 4000 V	
		Mobile phase: 0.1% formic acid in water and 0.1% formic acid in methanol	Inlet pressure: 30 psi and temperature 270 °C	
		Flow rate: 0.5 mL/min		
		Injection volume: 5 µL		
Protocatechuic acid; caffeic acid; vitexin; ferulic acid; taxifolin; trans-cinnamic acid; genistein	Sweet lupin seed	Column: Kinetex XB-C 18 (250 × 4.6 mm, 5 μm)	Nebulizing gas: nitrogen at 45 psi, 300 °C, and 5 L/min	[49]
		Column temperature: 25 °C	Capillary voltage: 3.5 kV	
		Flow rate: 0.5 mL/min	Nozzle voltage: −500 V	
		Mobile phase: 0.05% formic acid in water and acetonitrile	Sheath gas: nitrogen at 11 L/min and 250 °C	
		Injection volume: 20 µL		
Hesperetin; quercetin; naringenin; benzoic acid; naringin; narirutin; hesperidin; caffeic acid; neohesperidin; pinocembrin; taxifolin; fisetin; glabridin; eriocitrin; eriodictyol; formononetin; liquiritin; liquiritigenin; 3-hydroxybenzoic acid; 3,4-dihydroxybenzoic acid; 3-(4-hydroxyphenyl)propionic acid; 4-hydroxybenzoic acid; 3,4-dihydroxy-phenylacetichippuric acid; α-hydroxyhippuric acid; 3-hydroxyphenylacetic acid; p-coumaric acid; ferulic acid; and 4-hydroxy-3-methoxyphenylacetic acid	Plant materials	Column: Zorbax Eclipse XDB-C18 column (50 × 2.1 mm, 1.8 μm)	Capillary voltage: −4500 V	[31]
		Column temperature: 30 °C	Temperature: 500 °C	
		Mobile phase: 0.1% *v*/*v* formic acid in water and acetonitrile	Nebulizer gas: 60 psi	
		Flow rate: 0.5 mL/min	Turbo-gas: 50 psi	
		Injection volume: 5 µL	Collision activated dissociation gas: 4 psi	
			Curtain gas: 20 psi	
Eriocitrin; taxifolin; naringin; hesperidin; neohesperidin; fisetin; eriodictyol; naringenin; hesperetin; kaempferol; chrysin; glabridin	Commercial herbal dietary supplements	Fusion-RP XDB-C18 (50 × 2.0 mm, 4 μm)	-	[38]
		Column temperature: 30 °C		
		Mobile phase: 0.1% formic acid in water and acetonitrile		
		Flow rate: 0.3 mL/min		
		Injection volume: 2 µL		
Phloroglucinol; gallic acid; cyanidin 3-glucoside; chlorogenic acid, rutin; quercetin	Brown seaweed	Column: Atlantis C18 (250 × 4.6 mm, 5 µm)	Capillary voltage: 4000 V	[2]
		Column temperature: 25 ℃	Gas nebulizer: nitrogen	
		Mobile phase: 0.25% aqueous acetic acid and acetonitrile/water (80/20 *v*/*v*)	Pressure gas: 50 psi	
		Flow rate: 1.0 mL/min	Flow rate: 10 L/min	
		Injection volume: 10 µL	Drying temperature: 350 °C	
Gallic acid; catechin; caffeic acid; rutin; ferulic acid; quercitrin; resveratrol	*Connarus perrottetti* var. *angustifolius*, *Cecropia obtusa*, *Cecropia palmata*, and *Mansoa alliacea*	Column: C18 (250 × 4.6 mm, 5 μm)	Capillary voltage: ±2.4 kV	[28]
		Column temperature: 21 °C	Gas flow: 11 L/min	
		Mobile phase: orthophosphoric acid solution (0.1%, *w*/*w*) and acetonitrile	Nebulizer: 30 psi	
		Flow rate: 0.8 mL/min	Gas temperature: 250 °C	
			Drying gas: nitrogen	
Cis- and trans- resveratrol-3-O-galloylglucoside; methyl-(S)-flavogallonate; quercetin-7-O-di-glucoside; quercetin-7-O-galloyl-glucoside; naringenin-40-methoxy-7-pyranoside; 5,6-dihydroxy-30,40,7-tri-methoxy flavone; terminalin; corilagin derivative; oleanane type triterpenoids	*Terminalia brownii* (Fresen)	Column: Varian LC–18 (250 × 4.6 mm; 5 µm)	Spray voltage: 5000 V	[76]
		Column temperature: 30 °C	Capillary temperature: 280 °C	
		Mobile phase: acetonitrile and water containing 0.005% formic acid, acetonitrile, and glacial acetic acid	Sheathing gas: nitrogen at 40 U	
		Flow rate: 0.5 mL/min	Collision gas: helium at 0.8 mTorr	
		Injection volume: 5 µL		
Gallocatechin; epigallocatechin; catechin; epicatechin; epigallocatechin gallate; gallocatechin gallate; epicatechin gallate; catechin gallate; theaflavin; theaflavin-3-gallate	Tea	Column: Capcellpak C18 MGIII (100 × 2.0 mm, 3 µm)	Nebulizer gas flow: 60 mL/min.	[54]
		Column temperature: 30 °C	Cone temperature: 200 °C	
		Mobile phase: 0.1% aqueous formic acid and methanol	Cone gas flow: 20 mL/min	
		Flow rate: 0.3 mL/ min	Heated probe temperature: 300 °C.	
		Injection volume: 2 µL		
Oleuropein; acteoside; rutin	*Syringa vulgaris* L. flowers and fruits	Column: Zorbax SB-C18 (150 × 3.0 mm, 3.5 μm)	Capillary voltage: 3500 V	[37]
		Column temperature: 25 °C	Nebulizing and drying gas: nitrogen	
		0.1% (*v*/*v*) formic acid and methanol	Nebulizing gas pressure: 45 psi	
		Flow rate: 0.7 mL/min	Drying gas flow and temperature: 10 L/min and 300 °C	
			Fragmentor voltage: 170 V	
			Nozzle voltage: 500 V	
			Sheath gas flow and temperature: 10 L/min and 300 °C	
Gallic acid; protocatechuic acid; p-Hydroxybenzoic acid; vanillic acid; caffeic acid; syringic acid; p-coumaric acid; ferulic acid; rutin; quercetin-3-O-glucoside; quercetin; cyanidin-3,5-O-diglucoside; cyanidin-3-O-glucoside; cyanidin-3-O-rutinoside; eonidin-3-O-glucoside	Black rice wine	Column: SHIM-PACK XR-ODS (75 × 3.0 mm, 2.2 μm)	Ion spray voltage: 4400 and –4400 V	[29]
		Column temperature: 30 °C	Curtain gas (CUR): nitrogen	
		Mobile phase: 50% aqueous acetonitrile (*v*/*v*) with 0.2% formic acid and water with 0.2% formic acid	Nebulizer gas: air at 50 psi	
		Flow rate: 0.3 mL/min	Heater gas: air at 50 psi	
		Injection volume: 20 µL		
Apigenin; Baicalein; chrysin; p-coumaric acid; dihydroxytetramethoxy-flavone; dihydroxytrimethoxy-flavanone; eriodictyol; luteolin; naringenin; norwogonin; oroxylin a; pentahydroxyflavanone; pinocembrin; quercetin; scutellarein; sinapic acid; verbascoside; wogonin		Column: Zorbax Stable Bond Analytical SB-C18 column (250 × 4.6 mm, 5 μm)	Nebulizing and drying gas: nitrogen at 45 psi	[3]
		Column temperature: 35 °C	Electron spray voltage: 5.2 kV	
		Mobile phase: 0.1% aqueous formic acid and methanol	Source temperature at 500 °C	
		Flow rate: 0.5 mL/min		
		Injection volume: 10 µL		

In columns with non-modified alkyl chains, such as the C18 columns, the phenolic compounds are eluted according to their polarity. Generally, the phenolic compounds separation is carried out by gradient elution using binary systems comprising an aqueous component and a less polar organic solvent such as methanol or acetonitrile. Furthermore, with the aim to control pH in order to control the charge of the molecule, acids such as formic [1,33,77], acetic [2,54], or phosphoric [28] are normally incorporated in low percentages, between 0.005% and 0.5% (*v*/*v*) in the aqueous phase or even in both phases. Despite not being frequent, because silica-based columns can be irreversibly damaged at very low pH, some authors use higher percentages of acid (10%) [32]. Additionally, because the phosphoric acid is non-volatile, its use in mass spectrometer detection is not recommended. Acid pH between 2 and 4, contribute to avoiding phenolic compounds dissociation, help with defining peaks and improving the ionization efficiency for mass characterization [71,78]. Tong et al. [79] optimized different concentrations of acetic or formic acid in two mobile phases (water-methanol or water-acetonitrile) and also various gradient programs to determine several polyphenols in *Citrus paradisi* cv. *Changshanhuyu* peel. These authors found that the addition of 0.4% formic acid in the aqueous phase improved the polyphenol determination.

The selection of the flow rates and injection volume usually varies depending on the chosen column. As can be seen in Table 2, for polyphenols identification by LC–MS/MS the flow rate ranged between 0.2 and 0.8 mL/min and the injection volume from 2 to 40 μL.

Diode array detection (DAD) is the more used detector to quantify and identify polyphenols since it is cheap and robust. However, the identification and quantification of polyphenols is really complex largely due to the complexity of the plant material samples and the low concentrations in which they can be found. Although many standards are available, it is difficult to choose the correct standards, and the researchers must know in advance the components that the samples contain, to make a good selection of the standards. Additionally, the DAD identification is by retention time and by UV-vis spectrum. The polyphenols are linked to sugars that are not UV-active and hence will not affect the spectrum, which complicates correct polyphenol identification. Considering these difficulties, in many cases, it is necessary to use a more sensitive and selective detector such as a mass spectrometer to a LC system (LC–MS) or to a tandem mass spectrometer (LC–MS/MS). In some cases, the use of single quadrupole mass spectrometer is not selective enough for target compounds. In these cases, the use of a tandem mass spectrometer is necessary. Tandem mass spectrometers consist of three quadrupoles in which the first (Q1) and third quadrupole (Q3) are mass filters and the second quadrupole (Q2) acts as a collision cell. Thus, in comparison with a single quadrupole mass spectrometer, the presence of three quadrupoles make the spectrometer more selective, reduce signal-to-noise (S/N), present a wider linear range of quantitation, better accuracy, and reproducibility. Additionally, the identification of analytes is more real since it is able to use the multiple reaction monitoring (MRM).

Despite the differences and advantages reported for tandem mass spectrometers, multiple types of mass spectrometers can be used for polyphenol analysis, such as quadrupole (single or triple) [49,50], ion trap mass spectrometer [80,81,82], time-of flight or quadrupole-time-of-flight [10,79,80,83], and Orbitrap [33] among others. In the consulted literature there are studies demonstrating efficacy in polyphenols detection and quantification with different mass analyzers (Table 3).

Among tandem mass spectrometers, QqQ-MS presented high selectivity and sensitivity, but it is limited to structural characterization of non-target compounds [83]. Ion trap-MS is a good tool for the identification of unknown compounds, but the co-extracted ions can make correct selection of the diagnostic ions difficult. Finally, a QTOF-MS spectrometer offers accurate mass measurement, permitting better capability of identifying unknown chemicals than QqQ-MS and Ion trap-MS. Therefore, it seems clear that each of these analyzers have certain advantages and disadvantages compared to the others. Despite this, the use of tandem mass spectrometry is the most versatile tool for determining and quantifying polyphenols.

**Table 3:** Ionization mode, collision energy, and multiple reaction monitoring (MRM) transitions used in the polyphenol determination.

Analyte	Analyzer/Ionization Mode	Precursor Ion (m/z)	Product Ion (m/z)	Ref
3-(3,4-Dihydroxyphenyl)propionic acid	QqQ/ESI (−)	181	137	[34]
3-(3-Hydroxyphenyl)propionic acid	QqQ/ESI (−)	165	121	[34]
3-(4-hydroxy)phenylpropionic acid	QqQ/ESI (−)	165	121	[34]
3-(4-hydroxyphenyl)propionic acid	QqQ/ESI (−)	164.9	120.5	[31]
3,4-Dicaffeoylquinic acid	QqQ/ESI (−)	515	353, 235,191, 179, 173, 135	[51]
3,4-Dihydroxybenzoic acid	QqQ/ESI (−)	152.9	108.9	[31]
3,4-Dihydroxy-phenylacetic acid	QqQ/ESI (−)	166.9	122.7	[31]
3-Hydroxybenzoic acid	QqQ/ESI (−)	137	93	[31,34]
3-Hydroxyphenylacetic acid	QqQ/ESI (−)	150.9	107.0	[31]
3-Methoxyphenylacetic acid	QqQ/ESI (−)	180.8	136.8	[31]
3-*O*-Caffeoylquinic acid	QqQ/ESI (−)	353	191, 173, 85	[51]
3-*O*-methylquercetin	QTOF/ESI (−)	315	151, 271	[10]
4,5-Dicaffeoylquinic acid	QqQ/ESI (−)	515	353, 191, 179, 173, 135	[51]
4′,7′-Dimethoxy luteolin	QqQ/ESI (−)	313	298, 283, 255, 163, 117	[51]
4-Hydroxybenzoic acid	QqQ/ESI (−)	136.9	93.0	[31]
4-O-Feruloylquinic acid	QqQ/ESI (−)	367	191, 173, 134, 93, 87	[51]
	Qtrap/ESI (−)		193, 191, 173	[52]
5-(3,4-Dihydroxyphenyl)-γ-valerolactone	QqQ/ESI (−)	207	85	[34]
5-(3,4-Dihydroxyphenyl)-γ-valerolactone glucuronide	QqQ/ESI (−)	383	207	[34]
5,6,7,30,40-Pentahydroxyflavanon	Q-Trap/ESI (−)	479	303, 285, 181, 167, 135	[3]
5-*O*-Caffeoylquinic acid	QqQ/ESI (−)	353	191, 179, 173	[51]
5-*O*-*p*-Coumaroylquinic acid	Qtrap/ESI (−)	337	191,173	[52]
7-*O*-glucoronide	Q-Trap/ESI (−)	480	303, 285, 181, 167, 136	[3]
Acteoside	QqQ/ESI (−)	623.2	160.9	[37]
Apigenin-7-*O*-β-apiofuranosyl-6,8-di-C– β-glucopyranoside	QqQ/ESI (−)	725	635, 605, 593, 575, 503	[49]
Aromadendrin-6-C-β-D-glucopyranosyl-7-*O*-[β-D-apiofuranosyl-(1→2)]-*O*-β-D-glucopyranoside	QqQ/ESI (−)	743	653, 623, 581, 563	[49]
Astragalin	QqQ/ESI (−)	447.09	284.0	[33,50]
Avicularin	QqQ/ESI (−)	433.08	301.0	[33]
Benzoic acid	QqQ/ESI (−)	121	77	[31,34]
Caffeic acid	QqQ/ESI (−)	179	135	[1,2,29,31,34,36,50,84]
			135, 107, 89	[51]
Catechin	QqQ/ESI (−)	289.1	109.20	[50]
			245.1	[1]
			203	[34]
Catechin glucuronide	QqQ/ESI (−)	465	289	[34]
Catechin	QqQ/ESI (−)	289.1	245.1	[84]
Chlorogenic acid	QqQ/ESI (−)	353.1	191.1	[1,2,50]
			79, 191	[36]
Chrysin	QqQ/ESI (−)	252.9	143.0	[31]
	Qtrap/ESI (−)			[38]
Cinnamic acid glucoside	QqQ/ESI (−)	309	291, 247, 180, 128	[49]
Coumarin glycoside ester	Qtrap/ESI (−)	351	307, 145	[52]
Cyanidin-3,5-O-diglucoside	QqQ/ESI (+)	611.4	287.2	[29]
Cyanidin-3-*O*-glucoside	Qtrap/ESI (+)	449.2	287.2	[32]
	QqQ/ESI (+)			[29]
Cyanidin-3-*O*-rutinoside	QqQ/ESI (+)	595.4	287.2	[29]
Cynaroside	QqQ/ESI (−)	446.90	285.10	[50]
Dicaffeoylquinic acid	QTOF/ESI (−)	515	353, 191, 179	[10]
	QqQ/ESI (−)		249, 179, 135	[49]
Dihydrocaffeic acid glucuronide	QqQ/ESI (−)	357	181	[34]
Dihydroferulic acid glucuronide	QqQ/ESI (−)	371	195	[34]
Dihydro-*p*-coumaric acid derivative	Qtrap/ESI (−)	415	385, 165	[52]
Dihydroxybenzoic acid	QqQ/ESI (−)	153.0	109.0	[1]
Ellagic acid	QqQ/ESI (−)	301	145	[33,50]
Epicatechin	QqQ/ESI (−)	289.1	109.2	[50]
Epicatechin derivative	Qtrap/ESI (−)	397	365, 289, 207, 151	[52]
Epicatechin glucuronide	QqQ/ESI (−)	465	289	[34]
Epicatechin	QqQ/ESI (−)	289	203	[34]
Epigallocatechin	QqQ/ESI (−)	305.1	125.0	[50]
	Qtrap/ESI (−)	305	305, 273, 179	[52]
Eriocitrin	QqQ/ESI (−)	595.2	286.9	[31]
	Qtrap/ESI (−)			[38]
Eriodictyol	QqQ/ESI (−)	287.0	150.7	[31]
	Qtrap/ESI (−)			[38]
Ferulic acid	QqQ/ESI (−)	193	134	[31,34,36,50,84]
			177.9	[29]
			149	[2]
Ferulic acid glucoside	QqQ/ESI (-)	355	193, 178, 134	[49]
Fisetin	QqQ/ESI (−)	284.9	134.8	[31]
	Qtrap/ESI (−)			[38]
Formononetin	QqQ/ESI (−)	266.9	251.8	[31]
Gallic acid	QqQ/ESI (−)	169	125	[1,2,29,33,34,50,84]
	Qtrap/ESI (−)			[52]
			169, 125, 97	[30]
Gallic acid glycoside	Qtrap/ESI (−)	331	169	[52]
Genistein	QqQ/ESI (−)	269	269, 195, 133	[49]
Gentisic acid	QqQ/ESI (−)	153.10	109.30	[50]
Glabridin	QqQ/ESI (−)	323.2	201.3	[31]
	Qtrap/ESI (−)			[38]
Hesperetin	QqQ/ESI (−)	300.9	163.7	[31]
	Qtrap/ESI (−)			[38]
Hesperidin	QqQ/ESI (−)	609.0	301	[1,31]
	Qtrap/ESI (−)			[38]
Hippuric acid	QqQ/ESI (−)	178	134	[31,34]
Homovanillic	QqQ/ESI (−)	181	163	[34]
Hyperoside	QqQ/ESI (−)	463.1	300.0	[33,50]
Isoquercitrin	QqQ/ESI (−)	463	300.0	[33]
	QTOF/ESI (-)		301, 151	[10]
Isorhamnetin	QqQ/ESI (−)	315.10	300.10	[50]
Juglanin	QqQ/ESI (−)	417.08	284.0	[33]
Kaempferol	QqQ/ESI (−)	285	93.1	[50]
			93.4	[1]
			239	[34]
	Qtrap/ESI (-)		150.7	[38]
	Q-trap/ (+)	287	287, 258, 165, 153, 121	[30]
Kaempferol 3-*O*-hexoside	Q-trap/ (-)	447	447, 285, 255	[30]
Kaempferol 3-*O*-pentoside	Q-trap/ (+)	419	419, 309, 287, 155	[30]
Liquiritigenin	QqQ/ESI (−)	255.1	118.7	[31]
Liquiritin	QqQ/ESI (−)	417.2	255.0	[31]
Luteolin	QqQ/ESI (−)	285.10	133.20	[50]
	QTOF/ESI (-)	285	217, 151	[10]
Methylcatechin	QqQ/ESI (−)	303	137	[34]
Methylcatechin glucuronide	QqQ/ESI (−)	479	303	[34]
Methylepicatechin glucuronide	QqQ/ESI (−)	479	303	[34]
Methylgallate	Qtrap/ESI (−)	183	169,125	[52]
Methylgallic acid	QqQ/ESI (−)	183	168	[34]
Myricetin	QqQ/ESI (−)	316.9	179	[2]
		317.0	151.0	[1]
Naringenin	QqQ/ESI (−)	271	151	[31,36,50]
	Qtrap/ESI (−)	270.9	118.7	[38]
Naringin	Qtrap/ESI (−)	579.2	270.9	[38]
	QqQ/ESI (−)	579.0	271.1	[1,31]
Narirutin	QqQ/ESI (−)	579.3	270.9	[31]
Neohesperidin	QqQ/ESI (−)	609.0	300.8	[31]
	Qtrap/ESI (−)			[38]
Nicotiflorin	QqQ/ESI (−)	593.00	285.00	[50]
Nodakenin	Q-trap/ESI(+)	409	409, 391, 353, 389, 247, 229, 203, 185	[30]
Oleuropein	QqQ/ESI (−)	539.2	275.1	[37]
*p*-Coumaric acid	QqQ/ESI (−)	163	119	[1,29,31,34,36,50]
*p*-coumaric acid glucoside	QqQ/ESI (−)	325	163, 119	[49]
Pelargonidin-3 rutinoside	Qtrap/ESI (+)	579.2	433.1, 271.1	[32]
Pelargonidin-3-glucoside	Qtrap/ESI (+)	433.2	271.6	[32]
Pelargonidin-acetylglucoside	Qtrap/ESI (+)	475.2	271.2	[32]
Pelargonidin-malonylrhamnoside	Qtrap/ESI (+)	503.2	271.1	[32]
Pelargonidin-succinyl-arabinoside or	Qtrap/ESI (+)	503.2	271.1	[32]
Pentahydroxyflavanone	Q-Trap/(-)	303	257, 219, 167, 141, 129, 113	[3]
Pentahydroxyflavone	Q-Trap/(+)	303	303, 285, 257, 247, 235, 229, 179, 165, 153, 149, 137, 127	[3]
Peonidin-3-O-glucoside	QqQ/ESI (+)	463.0	301.2	[29]
Phenylpropionic acid	QqQ/ESI (−)	149	105	[34]
Phloroglucinol	QqQ/ESI (−)	125	57	[34]
			97	[2]
*p*-Hydroxybenzoic acid	QqQ/ESI (−)	137	93	[29,50]
Pinocembrin	QqQ/ESI (−)	254.8	150.7	[31]
Pinoresinol rhamnoside	Qtrap/ESI (−)	503	357	[52]
Procyanidin dimer	QqQ/ESI (−)	557	425	[34]
Procyanidin dimer B1and B2	QqQ/ESI (−)	577.10	407.20	[50]
Procyanidin trimer C1	QqQ/ESI (−)	865.00	289.00	[50]
Protocatechuic acid	QqQ/ESI (−)	153	109.	[29,34,50]
			141, 109	[51]
			135, 109	[49]
	Q-trap/ (-)		153, 109, 108	[30]
Quercetin	QqQ/ESI (−)	301	151	[1,29,31,34,36,50]
			179	[2]
	QTOF/ESI (-)		151, 179, 121	[10]
	Q-trap/ (+)		301, 273, 179, 153	[30]
Quercetin 3-*O*-hexoside	Q-trap/ (−)	463	463, 301, 300, 283, 271, 255, 151	[30]
Quercetin 3-*O*-pentoside	Q-trap/ (−)	433	433, 300, 273, 271, 255, 179, 151	[30]
Quercetin derivative	Qtrap/ESI (−)	657	493, 327, 301, 255	[52]
Quercetin-3-O-glucoside	QqQ/ESI (−)	463.1	300.7	[29]
Quercetin-3-*O*-sophoroside	QqQ/ESI (−)	625.2	299.8	[33]
Quercetin-O-glucoside	QqQ/ESI (−)	463	301, 179, 151	[51]
Quercitrin	Qtrap/ESI (+)	447.0	301.0	[32,33,84]
	QqQ/ESI (−)			
Quinic acid butyl ester	Qtrap/ESI (−)	247	247, 191	[52]
Quinic acid derivative	QqQ/ESI (−)	405	191, 111	[49]
Quinic acid methyl ester	QqQ/ESI (−)	205	143, 129, 114	[51]
Resveratrol	QqQ/ESI (−)	227.1	143.1	[1,34]
Rutin	QqQ/ESI (−)	609.0	300.1	[1,29,31,36,37,50,51,84]
Sinapoyl hexoside	Q-Trap/(−)	385	223, 205, 190, 179, 175, 163	[3]
Sophoraflavonoloside	QqQ/ESI (−)	609.20	284.0	[33]
Syringic acid	QqQ/ESI (−)	197.0	181.9	[29]
Taxifolin	QqQ/ESI (−)	303.2	284.7	[31]
	Qtrap/ESI (−)			[38]
*trans*-Ferulic acid	QqQ/ESI (−)	193.2	134.0	[1]
Tricin	Qtrap/ESI (−)	329	329, 189, 137	[52]
Tricin *O*-(syringyl alcohol) ether O-hexoside	Qtrap/ESI (−)	659	497, 329	[52]
Valeric acid	QqQ/ESI (−)	225	163	[34]
Vanillic acid	QqQ/ESI (−)	167	108	[29,34]
Vanillin	QqQ/ESI (−)	151.10	136.20	[50]
Vicenin	QqQ/ESI (−)	593	503, 473, 383, 353, 297	[49]
α-Hydroxyhippuric acid	QqQ/ESI (−)	193.9	72.8	[31]

ESI: electrospray ionization; Q-trap: quadrupole ion trap; QqQ: triple quadrupole mass spectrometer; Q-TOF: quadrupole time of flight.

In this regard, Nijat et al. [33] combined the ultra-high performance liquid chromatography coupled to quadrupole-orbitrap high resolution mass spectrometry (UHPLC–Q–orbitrap–HRMS) and high performance liquid chromatography triple-quadrupole linear ion trap mass spectrometry (HPLC–QqQ–LITMS) to detect and quantify polyphenols in Meiguihua oral solution; Jin et al. [83] studied the identification of polyphenols in mulberry cultivars with both TOF/MS and QqQ–MS, while Quatrin et al. [80] reported the characterization and identification of tannins, flavonols, anthocyanins, and matrix-bound polyphenols from jaboticaba fruit peel with two different mass spectrometer analyzers (LC–TRAP–MS/MS and LC–Q–TOF–MS/MS), while the quantification was carried out using HPLC–DAD technique. For quantitative analysis, the use of triple quadrupole mass spectrometers (QqQ) is common, which are capable of performing multiple reaction monitoring (MRM) [84,85]. In fact, MRM allows enhanced sensitivity and selectivity.

On the other hand, although in liquid chromatography–tandem mass spectrometry (LC–MS/MS) other sources of ionization can be used, the electrospray ionization (ESI) is the most employed. To improve the sensitivity and minimize the matrix effects it is necessary optimize several MS/MS parameters such as capillary voltage, declustering potential, collision energy, and dwell times before analysis. It is also necessary to choose the ion mode between positive or negative. There are studies that investigated the presence of flavonoid and glycosides phenolic acids in Ajwa date fruits by LC–ESI–MS–MS in both modes [86]. However, as is shown in Table 3, negative ion mode is the most commonly used mode when analyzing phenolic compounds with the exception for anthocyanins for which both ionization modes have been commonly reported. Finally, a particular mention must be reserved to the so-called high-throughput targeted and untargeted metabolomics-based approaches, which have been widely used in the last years to characterize the different polyphenolic classes in several plant-foods for human nutrition [87,88,89,90]. In this regard, the metabolomic approaches have been very helpful in identifying and quantifying a specific set of metabolites in a sample, with several advantages, such as the absence of a sample purification step, thus contributing to the understanding of several factors affecting the phenolic profile of a sample under investigation.

Therefore, taking into account the great complexity and variety of phenolic compounds that may be present in the same food or plant extract, makes the use of LC–MS/MS essential. Furthermore, as commented above, specific parameters must be selected for each family of phenolic compounds for their detection with mass spectrometry. For this reason, this review presents the data from the most recent studies, in a comprehensive way, providing and simplifying the information of the great variety of works that exist in the literature.

## 4. Conclusions

Polyphenols are of great interest from the point of view of health and industry. However, their use depends largely on good characterization and quantification of the active compounds present in the extracts and plant material. There is not a common extraction method for all types of polyphenols because of the large number of existing phenolic compounds and the old techniques to quantify their content have serious limitations. Therefore, the development of new techniques that allowed a correct characterization of phenolic compounds became essential. Moreover, extraction, purification, and clean-up stages have a key role for obtaining reliable results. The use of liquid chromatography with tandem mass spectrometry (LC–MS/MS) reported good results with low quantification limits in the polyphenols analysis. However, there are several researchers that used different extraction, chromatographic, and mass spectrometer conditions. Therefore, the present review arises from the need to have the information in an organized and well-structured way, since this is vital when deciding the best technique to use.

As a general conclusion, the LC–MS/MS is the best and most powerful technique for the correct identification and quantification of polyphenols. However, the development of the analytical method depends largely on the matrix to be analyzed as well as on the phenolic compounds it contains. Therefore, the information provided by this review, focused on LC–MS/MS technique, allows the scientific community to have a global vision of the main parameters used by other authors in recent studies, both in extraction and clean-up procedures as well as the chromatographic and mass spectrometer conditions.
